# Metabolically active tumour volume segmentation from dynamic [^18^F]FLT PET studies in non-small cell lung cancer

**DOI:** 10.1186/s13550-015-0102-6

**Published:** 2015-04-23

**Authors:** Lieke L Hoyng, Virginie Frings, Otto S Hoekstra, Laura M Kenny, Eric O Aboagye, Ronald Boellaard

**Affiliations:** Department of Radiology & Nuclear Medicine, VU University Medical Center (VUmc), P.O. Box 7057, 1007 MB Amsterdam, The Netherlands; Comprehensive Cancer Imaging Centre, Imperial College London, Hammersmith Hospital, W12 0NN London, UK

**Keywords:** ^18^F*-FLT*, *Dynamic*, *PET*, *NSCLC*, *Kinetic filtering*, *Segmentation*

## Abstract

**Background:**

Positron emission tomography (PET) with ^18^F-3′-deoxy-3′-fluorothymidine ([^18^F]FLT) can be used to assess tumour proliferation. A kinetic-filtering (KF) classification algorithm has been suggested for segmentation of tumours in dynamic [^18^F]FLT PET data. The aim of the present study was to evaluate KF segmentation and its test-retest performance in [^18^F]FLT PET in non-small cell lung cancer (NSCLC) patients.

**Methods:**

Nine NSCLC patients underwent two 60-min dynamic [^18^F]FLT PET scans within 7 days prior to treatment. Dynamic scans were reconstructed with filtered back projection (FBP) as well as with ordered subsets expectation maximisation (OSEM). Twenty-eight lesions were identified by an experienced physician. Segmentation was performed using KF applied to the dynamic data set and a source-to-background corrected 50% threshold (A50%) was applied to the sum image of the last three frames (45- to 60-min p.i.). Furthermore, several adaptations of KF were tested. Both for KF and A50% test-retest (TRT) variability of metabolically active tumour volume and standard uptake value (SUV) were evaluated.

**Results:**

KF performed better on OSEM- than on FBP-reconstructed PET images. The original KF implementation segmented 15 out of 28 lesions, whereas A50% segmented each lesion. Adapted KF versions, however, were able to segment 26 out of 28 lesions. In the best performing adapted versions, metabolically active tumour volume and SUV TRT variability was similar to those of A50%. KF misclassified certain tumour areas as vertebrae or liver tissue, which was shown to be related to heterogeneous [^18^F]FLT uptake areas within the tumour.

**Conclusions:**

For [^18^F]FLT PET studies in NSCLC patients, KF and A50% show comparable tumour volume segmentation performance. The KF method needs, however, a site-specific optimisation. The A50% is therefore a good alternative for tumour segmentation in NSCLC [^18^F]FLT PET studies in multicentre studies. Yet, it was observed that KF has the potential to subsegment lesions in high and low proliferative areas.

## Background

In recent years, several studies demonstrated ^18^F-3′-deoxy-3′-fluorothymidine ([^18^F]FLT) as a useful positron emission tomography (PET) tracer for the prediction and monitoring of tumour response to chemotherapy [1-3]. Tumour tissues that show changes in [^18^F]FLT uptake within a week of therapy have a high likelihood of responding to treatment [4,5]. To evaluate changes in tracer uptake and viable lesion volume quantitatively, analysis requires tumour segmentation. Various manual and (semi-)automatic methods for tumour delineation have been proposed [6-8]. As manual tumour delineation is time consuming, requires expertise and is prone to observer variation, automatic tools are preferable. The performance of a (semi-)automatic tumour segmentation method in PET may vary per tracer, cancer type and lesion location. Physiological biodistribution and kinetic characteristics differ per tracer and per cancer type. The location of the lesion determines the contrast in tracer uptake between tumour and surrounding tissue: the target-to-background ratio [9]. [^18^F]FLT shows lower overall uptake in tumours and higher uptake in certain healthy tissues, such as liver and bone marrow than, for example, [^18^F]FDG (2-fluoro-2-deoxy-D-glucose). Therefore, [^18^F]FLT PET may require different (semi-)automatic tumour segmentation methods for tumour delineation than [^18^F]FDG PET.

Gray et al. [10] proposed a supervised classification method based on kinetic filtering (KF) [11]. In the KF method, the time activity curve (TAC) of each voxel is compared to the typical reference TAC of several representative tissues (liver, bone marrow, soft tissue, etc.). In breast cancer, Gray et al. reported significantly different results of standard uptake values (SUVs) based on KF and manual delineation.

For response-monitoring purposes, the repeatability of quantitative measures, which are used to characterize tracer uptake changes, needs to be known. Therefore, it is required to determine the test-retest (TRT) performance of the KF method in [^18^F]FLT PET studies and to compare it with other (existing) segmentation methods. Furthermore, it is of interest to explore the sensitivity of the supervised classification method to varying imaging protocols. Finally, the method has not yet been tested for the segmentation of lung tumours.

The current study examines the TRT performance of several implementations of the KF method for segmentation of lesions in non-small cell lung cancer (NSCLC) [^18^F]FLT PET scans. Apart from segmentation performance, the TRT variability of SUVs and metabolically active tumour volumes as defined with KF will be compared with those based on a (static) 50% background corrected relative threshold method.

Please note that segmented tumour volume in this article refers to the metabolically active tumour volume as derived with PET, not the anatomical volume.

## Methods

### Segmentation methods

In this paper, we compare the segmentation and repeatability of several adapted versions of the KF algorithm and a 50% background corrected isocontour method (A50%), as used by Frings et al. [12]. A short introduction of the original KF method and of the A50% method is given below in order to introduce some basic terminology used in the remainder of the paper.

#### Kinetic-filtering algorithm

The supervised segmentation method is described in Gray et al. [10]. The method compares for each voxel its TAC to several typical TACs of the most characteristic tissues (liver, vertebrae, tumour, heart, lung and background). These predefined TACs for various tissue types are called *kinetic classes* (KCs). KCs are derived from averaging tissue TACs from several dynamic [^18^F]FLT PET studies. These are then used to segment lesions in (other) dynamic [^18^F]FLT studies based on calculation of the Mahalanobis distance [13]. This is the distance between the voxel TAC and tissue class, weighted by the kinetic class standard deviations:

1 $$ {D}_{\mathrm{M}}=\sqrt{{\displaystyle \sum_{t=1}^N{\left(\frac{p_t-{\mu}_t}{\sigma_t}\right)}^2}} $$

where *D*_M_ is the Mahalanobis distance of voxel *p* to tissue class *M*, *N* is the number of time frames, *p*_*t*_ is the activity of the voxel at time frame *t*, and *μ*_*t*_ and *σ*_*t*_ are the activity of the class and the standard deviation at that time frame, respectively.

In order to obtain the same temporal sampling (frames) as that of the PET study, the KCs are resampled to those of the PET scan to be analysed. Gray et al. found that the algorithm did not distinguish properly between vertebrae and tumour tissue. Therefore, the algorithm reclassifies voxels assigned to the vertebra class as tumour tissue.

#### Segmentation based on A50%

A50% is a semi-automatic threshold technique. The threshold method locates the voxel with the highest uptake value near a user-indicated starting point in the tumour. Next, a region-growing algorithm using the location of the maximum tumour voxel value continues until all voxels above a certain threshold are included. In the case of the A50% method, this threshold is 50% of the sum of the maximum uptake value and the local background value. The local background value is calculated by averaging the values of a 1-voxel-thick shell at 1.5-cm distance from the boundary of an initial 70% of the maximum value isocontour. More details can be found in [14-16]. Frings et al. compared various threshold settings [12] in [^18^F]FLT PET and found that the 50% background adapted threshold, A50%, had the best trade-off between success rate and repeatability. Here, success rate is defined as the feasibility to define the tumour volume of interest (VOI).

### Clinical data sets and PET reconstruction

The present study involved three different KF reference data sets, each of which has been described in previous publications. The patients were included in the clinical studies after providing written informed consent in accordance with institutional review board approval. The first data set originates from the Hammersmith and Charing Cross Hospitals, London, United Kingdom, and was provided only in the form of tissue KC [10]. The other two data sets were collected at the VU University Medical Centre (VUmc), Amsterdam, The Netherlands [1,4]. Details of the data acquisition that are directly relevant for this paper are given below; further information can be found in the original publications of these studies.

Gray et al. [10] created tissue KC from the Hammersmith data. For the main part of our study, the KF method was implemented using these KCs. The Hammersmith data consisted of 13 [^18^F]FLT PET scans of patients with histologically proven stage II-IV breast cancer based on American Joint Committee on Cancer stage (AJCC) criteria. A median dose of 338 MBq [^18^F]FLT (range 151 to 381 MBq) was injected intravenously. Simultaneously with the injection, the emission scan was started using an ECAT962*/*HR+ PET scanner (CTI*/*Siemens, Knoxville, Tennessee, USA). The patients were scanned for 95 min, and the data were binned into 31 discrete time frames of varying duration (10 × 30 s, 5 × 60 s, 5 × 120 s, 5 × 180 s, 6 × 600 s). A subsequent transmission scan was used for attenuation correction. The images were reconstructed using the ordered subsets expectation maximisation (OSEM) method. The KC based on these data will be referred to as *Hammersmith KC*.

Alternative KCs were derived from one of the VUmc datasets to test whether different scanning protocols could affect the classes and the KF segmentation performance. The data consisted of nine [^18^F]FLT PET scans of patients with histological-confirmed NSCLC adenocarcinoma [1]. [^18^F]FLT (248 MBq, range 226 to 270 MBq) was injected intravenously 30 s after starting a dynamic emission scan in 3D setting on a ECAT EXACT HR+ scanner (Siemens/CTI, Knoxville, TN, USA). The total scan time was 60.5 min with the following frame lengths: 1 × 30 s, 6 × 5 s, 6 × 10 s, 3 × 20 s, 5 × 30 s, 5 × 60 s, 8 × 150 s and 6 × 300 s. All emission scans were reconstructed with 128 × 128 matrices using filtered back projection (FBP) with a Hanning filter (cut-off, 0.5 cycles per pixel). In addition, the images were reconstructed using OSEM, with 4 iterations and 16 subsets followed by post-smoothing of the reconstructed images using a 5-mm full width at half maximum (FWHM) Hanning filter. In the remainder of this paper, the kinetic classes based on this data set will be referred to as the *VUmc KC*.

The third data set was used to evaluate tumour segmentation performance of KF and A50%. The TRT study was first described in a previously published study [1]. The data consist of [^18^F]FLT PET scans of nine patients with histologically proven NSCLC. The patients were scanned twice within 7 days (mean 1.9 days, median 1 day) before any treatment. The patients were given an intravenous bolus injection with a median of 364 MBq [^18^F]FLT (range 252 to 397 MBq). Simultaneously, a 60-min PET scan was started using an ECAT962*/*HR+ PET scanner (CTI*/*Siemens, Knoxville, TN, USA). The 39 discrete time frames had varying durations (6 × 5 s, 6 × 10 s, 3 × 20 s, 5 × 30 s, 5 × 60 s, 8 × 150 s and 6 × 300 s). The scans were reconstructed both with FBP and OSEM in the same way as applied for obtaining the VUmc KC. From here on, this data set will be referred to by the *test-retest data* or *TRT data*.

### Kinetic classes of kinetic filters

KCs are derived as described in Gray et al. [10]. For every scan of the Hammersmith OSEM-reconstructed data, the average of the last three frames was created, and tissue VOI was defined manually by an experienced physician on the several tissue types. Next, dose-normalized TACs were extracted for every tissue type. The TACs were averaged per tissue type for all scans, resulting in a mean dose-normalized TAC or KC. The standard deviations for all time frames were obtained and used as weighting factors during KF segmentation, as described previously in the ‘Kinetic-filtering algorithm’ section (Equation 1). As indicated before, these reference tissue classes were provided to us and are called the *Hammersmith kinetic classes* (*Hammersmith KC*).

For the VUmc KF development dataset, the same procedure was applied to create VUmc-specific KC, both from the FBP- and the OSEM-reconstructed images. These KCs are called the *VUmc kinetic classes* (*VUmc KC*).

The TRT data were averaged over the last three frames (45- to 60-min post injection (p.i.)) for both FBP- and OSEM-reconstructed images. As no anatomical imaging data was available, VOIs were delineated manually from the averaged image data. A total of 28 tumour lesions were identified by an experienced physician. The VOIs were defined including the entire tumour plus a rim of a few voxels with soft tissue surrounding the lesions. These VOIs are used as *lesion masks*. As will be explained later, the lesion masks are used to generate some adapted versions of the KF method and to evaluate the performances of the different implementations of the KF method.

### KF method adaptations

During the evaluation of the KF methods using the VUmc TRT dataset, several adaptations of the KF method were explored. The adapted KF versions were kept identical to the original method as described in Gray et al. [10], except for the following changes:

- KF of FBP- versus OSEM-reconstructed TRT data.
- Use of different sets of KC: the Hammersmith classes and FBP-reconstructed and OSEM-reconstructed VUmc kinetic classes.
- Adjustment of weighting factors in calculating the Mahalanobis distance. The standard deviations were scaled with several values (0.5, 1.0 and 2.0).
- TRT data scans were resampled to better resemble the frame duration and distribution of the Hammersmith data. The total number of frames was reduced from 40 to 31 by reduction of the first 10-min data from 26 to 16 time frames. This was performed by temporally interpolating the TRT data to the Hammersmith frame times.
- Reclassification of voxels that are incorrectly classified as liver and/or vertebrae within the lesion mask into tumour voxels.
- Reduction of the set of KCs, by leaving out the liver KC or both the liver and vertebrae KC.
- Temporal smoothing of voxel TACs before application of the KF method. Every frame was averaged with the previous and the next time frames.
- Limitation of the time interval of the PET data. Tested time intervals are: 0 to 5, 9 to 60, 15 to 60 and 30 to 60 min. Frames outside the time interval were disregarded during classification.

Combinations of these adaptations were tested leading to over 50 different KF versions. In Table 1, a small subset of the most successful implementations is summarized.

**Table 1:** **Overview of various KF methods and their corresponding settings**

**KF version**	**KC**	**Description**	**Undetected lesions**
1	H’smith	Original KF algorithm	13
2	H’smith	Temporal smoothing was added	11
3	H’smith	Frame times of the scan data adjusted	2
4	H’smith	Liver KC reclassified as tumour KC, classification of 9- to 60-min p.i.	4
5	H’smith	Liver KC reclassified as tumour KC, classification of 20- to 60-min p.i.	2
6	H’smith	Liver KC omitted	6
7	H’smith	Liver and vertebrae KS omitted	1
8	VUmc	Original KF algorithm	10
9	VUmc	Liver KC reclassified as tumour KC, temporal smoothing, classification of 15- to 60-min p.i.	2

Only the results of the original KF and the eight most successful adaptations are provided. KC, kinetic class; KF, kinetic filtering; p.i., post injection.

### Performance evaluations

For each test and retest scan of the TRT dataset, (combinations of) the above-mentioned adapted versions of the KF method were applied to automatically segment the tumour. In addition, the A50% method was applied as reference semi-automated lesion delineation. The metabolically active tumour volume and SUV TRT (percentage difference and absolute differences) for both measures were compared using those lesions that were detected by both methods. The absolute difference and relative TRT variability for volume and SUV between test and retest data were calculated for both the KF and A50% methods for all identified lesions (within the manually predefined lesion masks) and averaged. The TRT variability was calculated as the absolute value of difference between the test and retest value, divided by the mean of both measurements and multiplied by 100. In order to further compare the A50% and KF segmentation performance, the number of lesions that could be reliably segmented by each method was reported.

## Results

**Figure 1:**
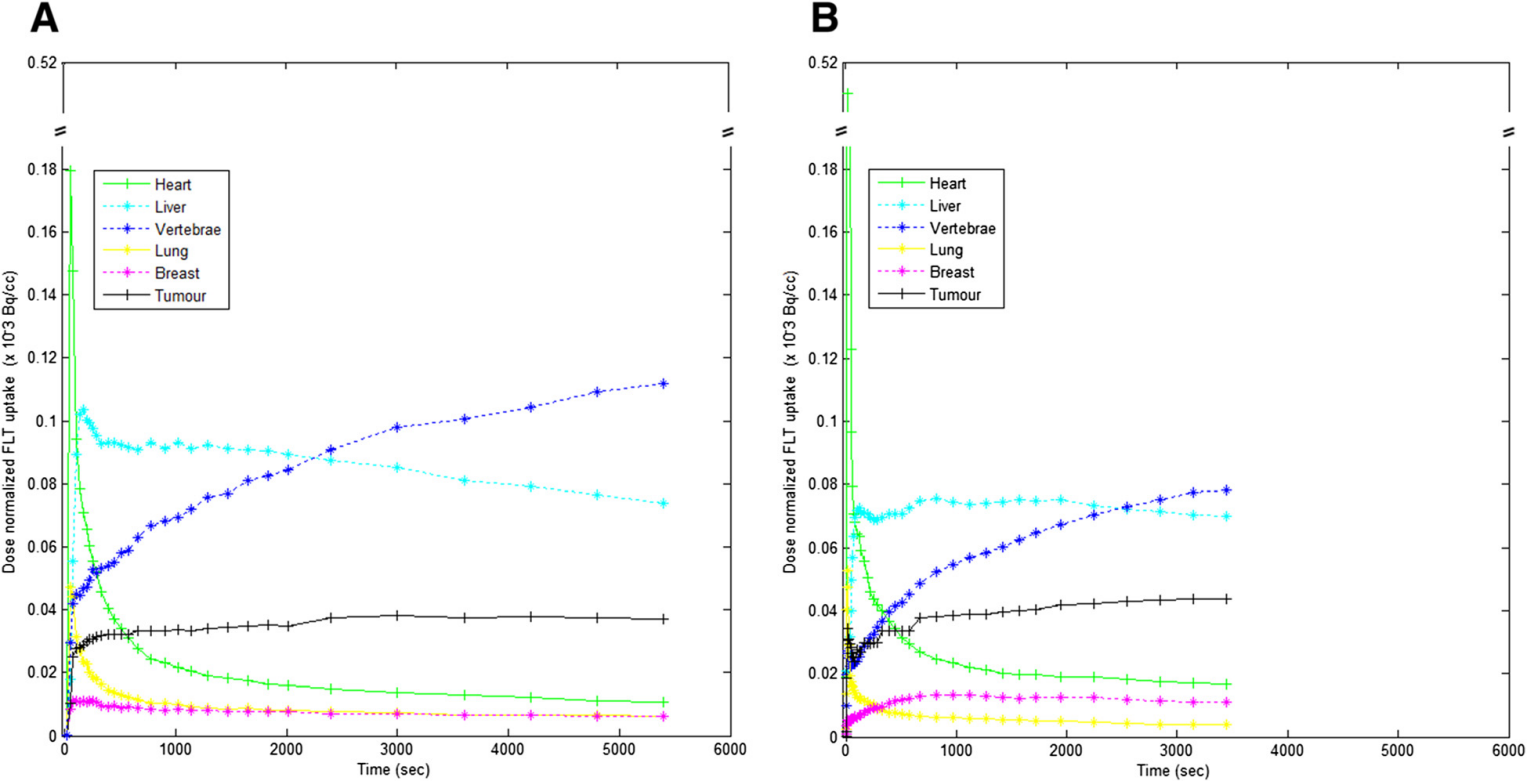
Hammersmith **(A)** and VUmc **(B)** kinetic classes.

The TACs of the Hammersmith and VUmc KC are shown in Figure 1. Though the shape of the KCs appear to be similar, differences can be observed in amplitude, standard deviations, frame distribution and total scan time (90 vs. 60 min for Hammersmith and VUmc scans, respectively).

**Figure 2:**
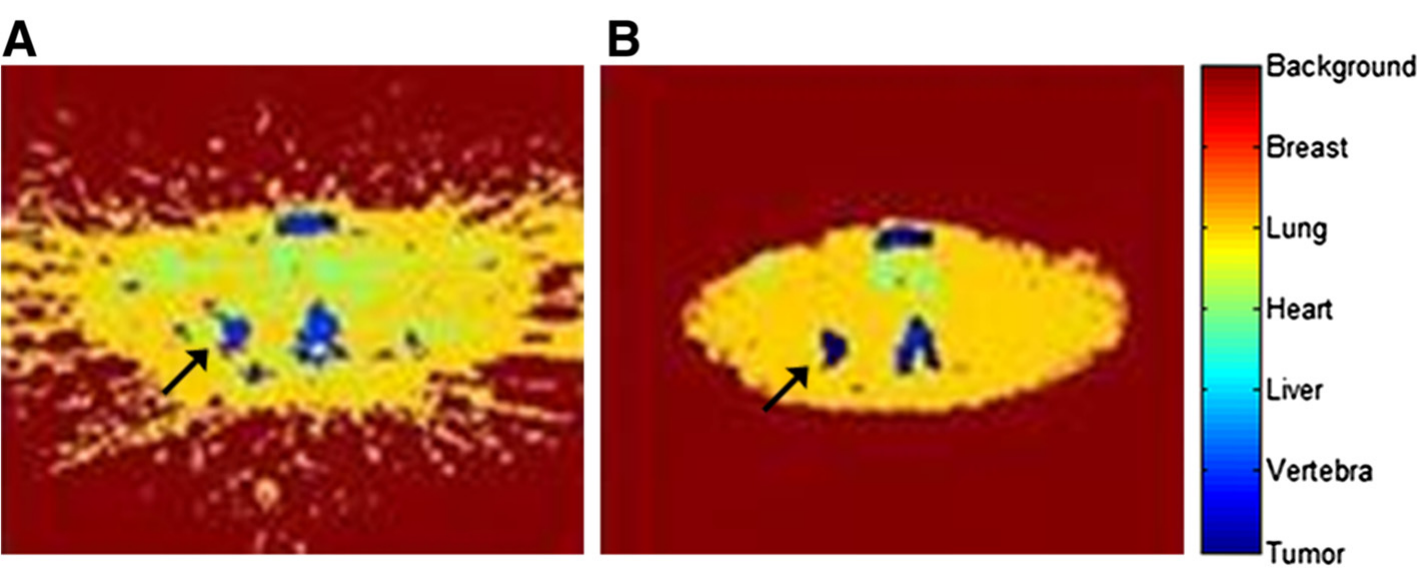
Example of KF classification. Example of a KF classification of an axial slice, FBP- **(A)** versus OSEM- **(B)** reconstructed image; the black arrow points at tumour segmentation from corresponding images shown in Figure 3.

**Figure 3:**
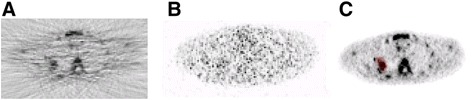
Example [^18^F]FLT PET axial slice, FBP and OSEM reconstructed. Axial slice showing [^18^F]FLT uptake, using FBP and OSEM reconstructions: FBP, 60-min p.i. **(A)**; OSEM, 5-min p.i. **(B)**; OSEM, 60-min p.i. The tumour has a coloured overlay of the lesion mask **(C)**.

The original as well as various modified versions of the KF method provided very poor segmentation results when applied to FBP-reconstructed images due to image artefacts. Figure 2A is an example of the result of post-processing a FBP-reconstructed image with the kinetic-filtering method. The artefacts that are visible on the original FBP image (Figure 3A) are clearly affecting the classification of the kinetic-filtering method (Figure 2A). Therefore, we only considered OSEM-reconstructed images of both KF and A50% (Figures 3B, C and 2B).

**Figure 4:**
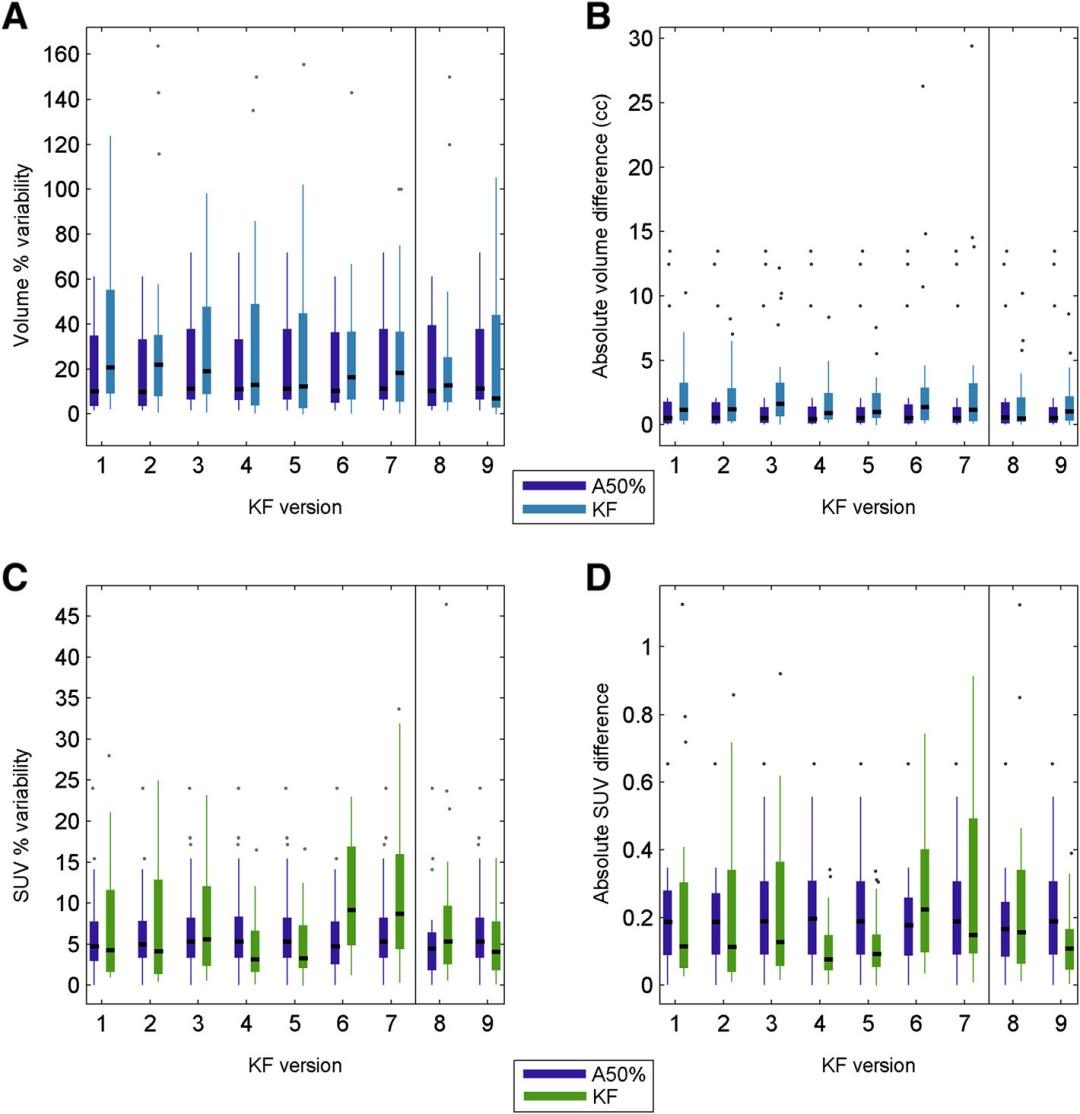
Test-retest performance of the most successful KF versions and of A50%. Percentage metabolically active tumour volume variability **(A)**, absolute metabolically active tumour volume difference **(B)**, percentage SUV variability **(C)** and absolute SUV difference **(D)**. Per KF version, the A50% TRT performance is shown for those lesions that were detected by the specific KF version. KF versions are listed in Table 1. KF, kinetic filtering; SUV, standard uptake value.

The median A50%-defined lesion size (PET volume) was 8.6 cm^3^ (range 0.7 to 51.9 cm^3^). The original implementation of KF segmented 15 out of 28 lesions (KF1, Table 1), whereas A50% segmented all lesions. All undetected lesions were metastases and had a A50%-defined metabolic volume smaller than 2.5 cm^3^. All primary lesions and metastases larger than 2.5 cm^3^ were identified. On average, the original implementation of KF (using the Hammersmith kinetic filters) provided 97% larger volumes and 16% lower SUV than measured with A50%. The lower SUV obtained with KF compared to A50% was caused by the larger tumour segmentation as well as by KF misclassification of highly active areas in the lung tumour as being liver, which were excluded from the obtained tumour segmentation. Median metabolically active tumour volume and SUV TRT variability of KF and A50% were 20.7% vs. 10.0% and 4.7% vs. 4.3%, respectively, as also illustrated in the boxplot in Figure 4A and C, respectively.

Given the high number of either undetected lesions or partly misclassified lesions by the original implementation of KF, our aim was primarily to optimize the KF method performance and test its feasibility for the VUmc TRT data. Due to the large amount of KF versions, only the results obtained from the original and the best performing implementations are shown in Figure 4 and listed in Table 1.

The best performing KF implementations in combination with the Hammersmith KC were those that were obtained using (in random order) the following: temporal smoothing (*KF2*); frame times of the TRT data PET scans interpolated to the frame times of the Hammersmith KCs (i.e. not vice versa, *KF3*); KC liver being reclassified as KC tumour in combination with analysis over a limited time interval from 9- to 60-min p.i. (*KF4*); KC liver being reclassified as KC tumour, analysis over a limited time interval from 20- to 60-min p.i. (*KF5*); use of a reduced set of KC, i.e. the liver KC was omitted (*KF6*); use of reduced set of KC, i.e. both liver and vertebrae KC were omitted (*KF7*); and the original KF implementation (*KF1*) in combination with the VUmc KC is indicated by *KF8*. The best performing KF implementation in combination with the VUmc KC was the one that reclassified liver and vertebrae as tumour KC, used temporal smoothing and restricted the analysis time interval from 15- to 60-min p.i. (*KF9*).

The adapted KF method versions (as listed above and in Table 1) reduced the amount of undetected lesions and improved the percentage repeatability performance as compared to the original KF implementation (*KF1*). The best performing KF methods in combination with the Hammersmith KC were *KF3*, *KF4* and *KF5*, i.e. when using temporally resampled PET images, reclassifying liver as tumour or when leaving out liver as a kinetic class.

**Figure 5:**
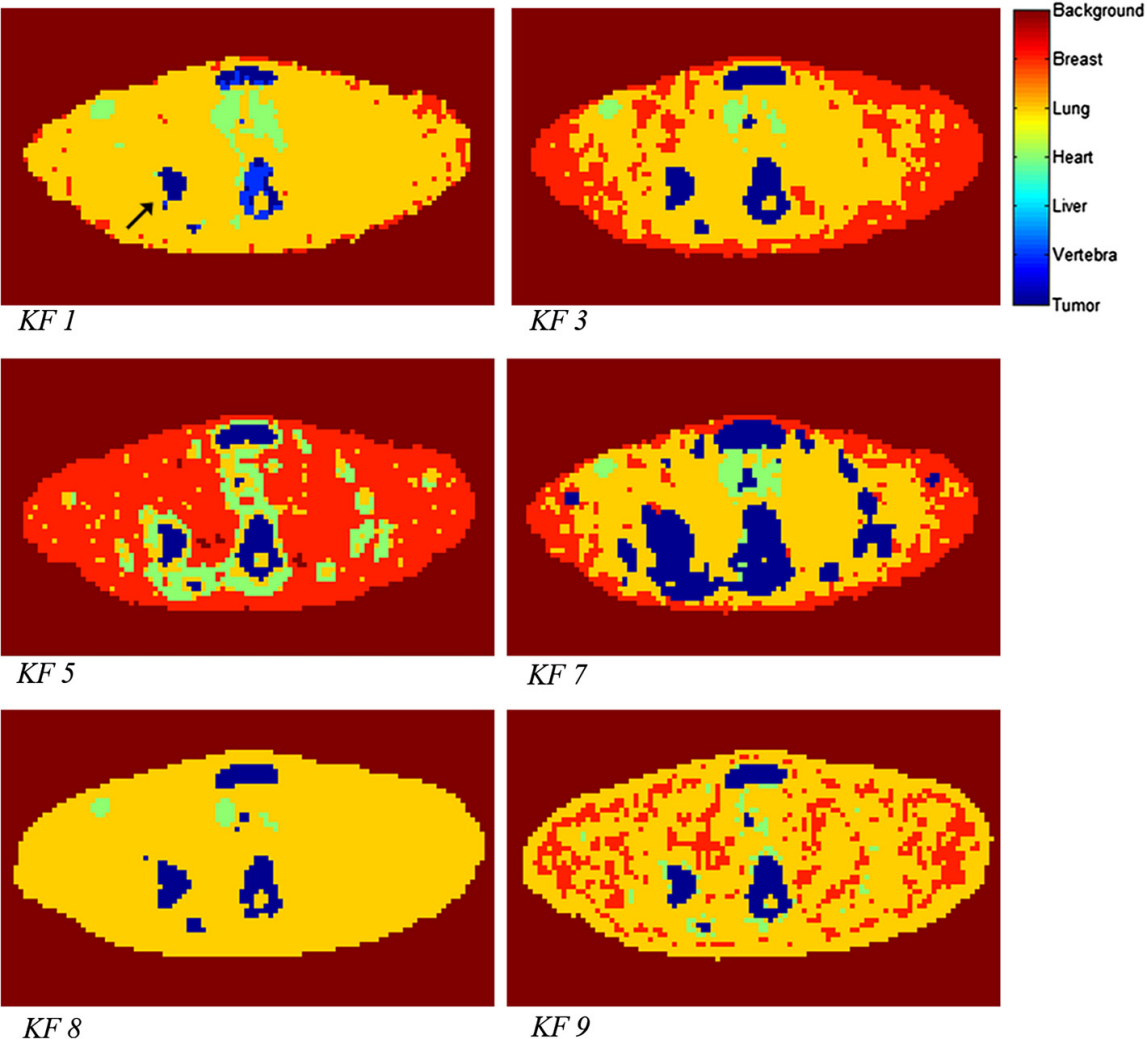
Segmentation examples obtained from several KF implementations. The KF numbering corresponds to the KF numbering as listed in Table 1. The black arrow in the image of KF1 points at the tumour. Every image represents the same axial image plane in the same patient. KF, kinetic filtering.

KF with temporal smoothing and a reduced set of KC (no liver and vertebral KC) (*KF7*) showed a good TRT variability compared to other KF implementations and was able to reliably segment all but one lesion. Figure 5 shows that the method classified too many voxels as tumour, even outside the lesion mask. This version of the KF method is therefore not successful. The red colour in the lung area in the segmentation pictures of *KF3*, *KF5* and *KF9* shows misclassification of heart and lung tissue as breast tissue. However, repeatability of metabolically active tumour volume was similar to that of A50% and fewer lesions were undetected in these versions as compared to the original KF. However, the spread in these methods is higher than with A50%.

**Figure 6:**
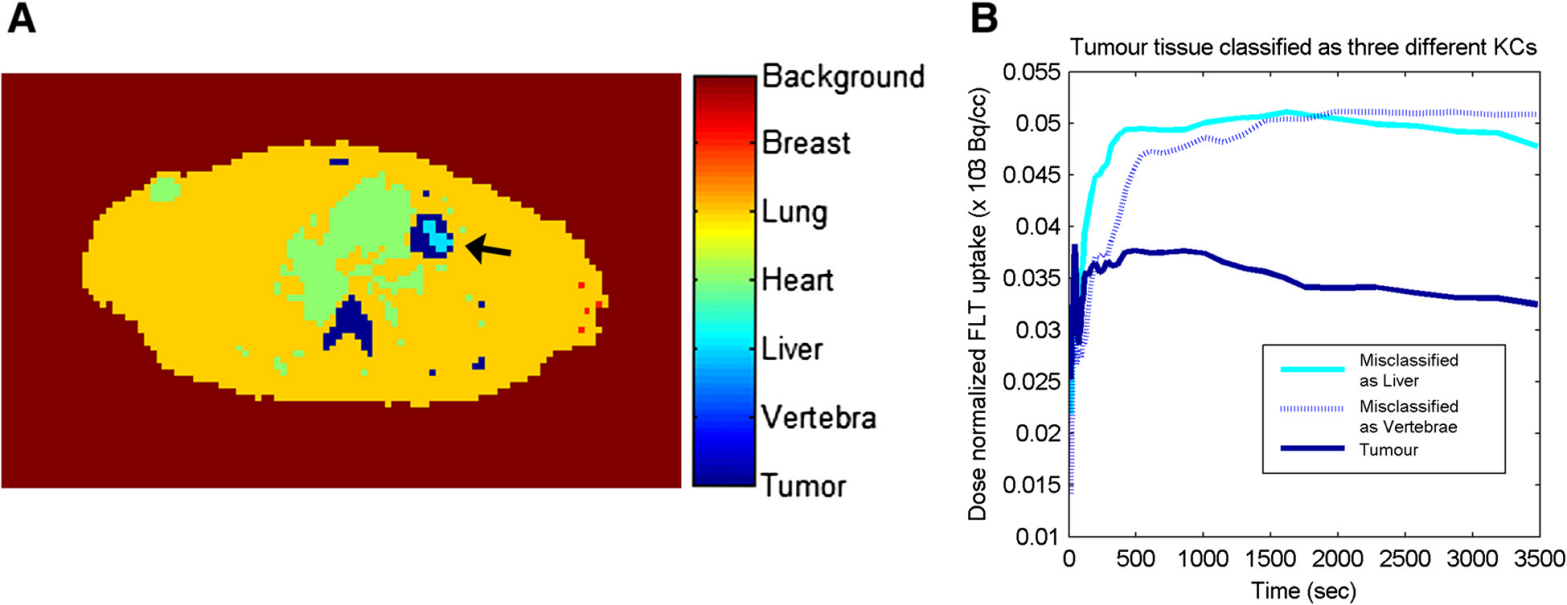
Misclassification of KF showing possibly heterogeneous tumour areas. The black arrow points at a tumour area that now encloses two different segmentations, KC tumour and KC liver, indicated by different colours **(A)**. The tumour part that is classified as liver and/or vertebrae indicates areas with a high proliferation rate **(B)**. KC, kinetic class.

Misclassification of tumour voxels as liver or vertebrae was observed in the original KF implementation. After further analysis, these misclassifications seem to reflect a heterogeneous kinetic behaviour within the tumour as shown by the high-uptake trend of the averaged TACs of the tumour voxels classified as liver or vertebrae (Figure 6B).

## Discussion

KF allows automated segmentation of lesions from dynamic [^18^F]FLT PET studies. In this study, various modifications of the KF method of Gray et al. [10] are examined. Since none of the tested methods reliably distinguished vertebrae and tumour, we introduced lesion masks. Combined with the KF method, these masks enable the segmentation of tumour as well as redefinition of voxels classified as liver and/or vertebrae into tumour (as there can be no liver or vertebrae within the lesion masks).

Comparison of the Hammersmith KC with the VUmc KC shows that the TACs of several KCs are very similar in shape and in relative uptake intensity (Figure 1 Hammersmith (A) and VUmc (B) kinetic classes). Tumour tissue and bone marrow show relatively high uptake due to rapid proliferation. The average [^18^F]FLT PET bone marrow TAC demonstrates irreversible tissue kinetics. The liver metabolizes [^18^F]FLT to [^18^F]FLT-glucuronide causing an even higher uptake signal in liver tissue but with explicit reversible kinetics, i.e. a decreasing trend over time. Recent studies suggest that most NSCLC tissue TACs are best fitted by a two-tissue reversible model in nonlinear regression analysis when applying a 90-min dynamic scan [17-19]. Small amplitude and shape discrepancies between the two KCs might be caused by a difference in tracer injection and/or imaging procedure. Differences in administration procedures, i.e. a fast or slow bolus injection, have a direct effect on the shape of any TAC for all tissues which may also affect the performance of the KF methods. As the use of Hammersmith KC versus VUmc KC has a large effect on the observed TRT performances, use of optimized or site-specific KC seems to be important for obtaining reliable results. Consequently, application of KF to multicentre studies requires highly standardized imaging procedures to apply KC derived elsewhere, or alternatively, the KCs need to be defined per imaging site. Furthermore, the specific framing of the dynamic scan affects KF performance. Best results with Hammersmith KC were obtained by either adjusting the frame times to a more even distribution of the time frames over the course of the PET scan or by disregarding the first 10 min of the scan (KF3-KF5). The 30-min difference in total scan duration between the two sites might be relevant in this context as well. Better TRT performance was obtained by allowing a more prominent role of the ‘tails’ of the TACs in the distance calculations. Possibly, use of longer than 60-min scan durations could have further improved performance; however, this was not possible with the currently available datasets. Optimisation of administration procedure in combination with adjustment of frame times of the dynamic scans might improve the performance of the tested KF methods. Moreover, we found a difference in the standard deviations of the Hammersmith KC and VUmc KC. Since these act as the weighting factor when calculating the Mahalanobis distance, this difference can influence the classification results. Although we tested various levels of rescaling of the amplitude of the weighting factors, we did not find an optimal setting. Reclassification of voxels that were initially classified as vertebrae or liver to tumour provided the best TRT results (KF4 and KF5) with Hammersmith KC. Obviously, when studying liver metastases, this would not be appropriate. Similarly, we expect the A50% isocontour method to be less reliable in hepatic metastases due to the high physiological [^18^F]FLT uptake value of the liver. KF with VUmc KC needed adjustment as well due to the required order of the Mahalanobis distances between a voxel and the KCs to allow for KC reclassification of KC vertebrae. Reduced VUmc KC performed best with only 2 missing lesions out of 28 and good metabolic active tumour volume and SUV TRT variability. Currently, patients with liver metastases are undergoing [^18^F]FLT-PET scans in order to further assess the performance of KF in areas with high physiological [^18^F]FLT uptake in a future study.

Scan and administration procedures were identical for both the VUmc development dataset and the TRT dataset, and therefore, obviously KF performed better in combination with the VUmc KC than in combination with the Hammersmith KC. If KC were not adapted to the study-specific and centre-specific tracer administration and scanning settings, then 50% of the lesions were not properly segmented. Yet, after carefully adjusting the method to align KC to scan conditions of the TRT data set or applying KC derived from scans performed on the same scanner and obtained using the same imaging procedure, the KF method performance improved and was able to reliably delineate 93% of the lesions. At the same time, metabolically active tumour volume and SUV TRT performances improved to similar levels observed with the A50% method (around 10%, on average). The improved performance after local (study-specific) optimisation of KF again illustrates that for a multicentre study standardized administration and scanning protocols are essential. Yet, once KF is optimized to the specific scan conditions at hand, the method can be used as an initial automated visualisation to identify lesions in dynamic [^18^F]FLT PET studies.

An additional advantage of KF classifications could be their potential use as a segmentation method for kinetically heterogeneous tumours. Detection of tumour heterogeneity could be used as a prognostic biomarker, to asses treatment response and for dose escalation in radiation therapy [20-22]. Therefore, it is necessary to study (automatic) delineation of intratumour subvolumes. To date, only few such studies have been performed to address this issue [9,23]. In our study, the observed intratumour (mis-)classification of voxels into liver or vertebrae may indicate that the method can differentiate between metabolically high and low active tumour tissues within a predefined lesion mask. Some preliminary results are shown in Figure 6, together with TACs, for these differently classified intratumour regions (now still denoted as liver and vertebrae). Further adaptations of this method might improve its use for intratumour tracer uptake heterogeneity assessment or segmentations and will be part of future research.

## Conclusions

For dynamic [^18^F]FLT PET studies in NSCLC patients, kinetic filtering and an adaptive contrast-oriented isocontour method (A50%) have similar repeatability performance to define metabolically active tumour volume but only after careful optimisation of the kinetic-filtering method settings. The kinetic classes and/or the kinetic-filtering method therefore requires a protocol-specific optimisation. In multicentre studies, A50% might be a good alternative segmentation method for [^18^F]FLT PET in NSCLC. Yet, KF has the potential to identify low and high proliferative areas within heterogeneous lesions.
